# An Allosteric Signaling Pathway of Human 3-Phosphoglycerate Kinase from Force Distribution Analysis

**DOI:** 10.1371/journal.pcbi.1003444

**Published:** 2014-01-23

**Authors:** Zoltan Palmai, Christian Seifert, Frauke Gräter, Erika Balog

**Affiliations:** 1 Department of Biophysics and Radiation Biology, Semmelweis University, Budapest, Hungary; 2 Molecular Biomechanics, Heidelberger Institut für Theoretische Studien gGmbH, Heidelberg, Germany; 3 MPG-CAS Partner Institute and Key Laboratory for Computational Biology, Shanghai, China; Fudan University, China

## Abstract

3-Phosphogycerate kinase (PGK) is a two domain enzyme, which transfers a phosphate group between its two substrates, 1,3-bisphosphoglycerate bound to the N-domain and ADP bound to the C-domain. Indispensable for the phosphoryl transfer reaction is a large conformational change from an inactive open to an active closed conformation via a hinge motion that should bring substrates into close proximity. The allosteric pathway resulting in the active closed conformation has only been partially uncovered. Using Molecular Dynamics simulations combined with Force Distribution Analysis (FDA), we describe an allosteric pathway, which connects the substrate binding sites to the interdomain hinge region. Glu192 of alpha-helix 7 and Gly394 of loop L14 act as hinge points, at which these two secondary structure elements straighten, thereby moving the substrate-binding domains towards each other. The long-range allosteric pathway regulating hPGK catalytic activity, which is partially validated and can be further tested by mutagenesis, highlights the virtue of monitoring internal forces to reveal signal propagation, even if only minor conformational distortions, such as helix bending, initiate the large functional rearrangement of the macromolecule.

## Author Summary

3-Phosphoglycerate kinase (PGK) is an essential enzyme for living organisms. It catalyzes the phospho-transfer reaction between two catabolites during carbohydrate metabolism. In addition to this physiological role, human PGK has been shown to phosphorylate L-nucleoside analogues, potential drugs against viral infection and cancer. PGK is a two domain enzyme, with the two substrates bound to the two separate domains. In order to perform its function the enzyme has to undergo a large conformational change involving a hinge bending to bring the substrates into close proximity. The allosteric pathway from the open non-reactive state of PGK to the closed reactive state as triggered by substrate binding has only been partially uncovered by experimental studies. Here we describe a complete allosteric pathway, which connects the substrate binding sites to the interdomain hinge region using Molecular Dynamics simulations combined with Force Distribution Analysis (FDA). While previously identified key residues involved in PGK domain closure are part of this pathway, we here fill the numerous gaps in the pathway by identifying newly uncovered residues and interesting candidates for future mutational studies.

## Introduction

3-Phosphoglycerate kinase (PGK) is a key enzyme in glycolysis that catalyzes phospho-transfer from 1,3-bisphosphoglycerate (BPG) to ADP producing 3-phosphoglycerate (PG) and ATP [1]. It has been shown that human PGK (hPGK) also phosphorylates L-nucleoside analogues, which are potential antiviral and anticancer drugs [2]–[6]. PGK is a monomeric enzyme composed of two domains of approximately equal size with the C-terminus of the protein bending back to the N-terminal domain, constituting an integral part of it. BPG binds to the N-terminal domain while the ADP binding site is located on the C-terminal domain (for the nomenclature of the secondary structure elements see Suppl. Table S1.)

**Figure 1:**
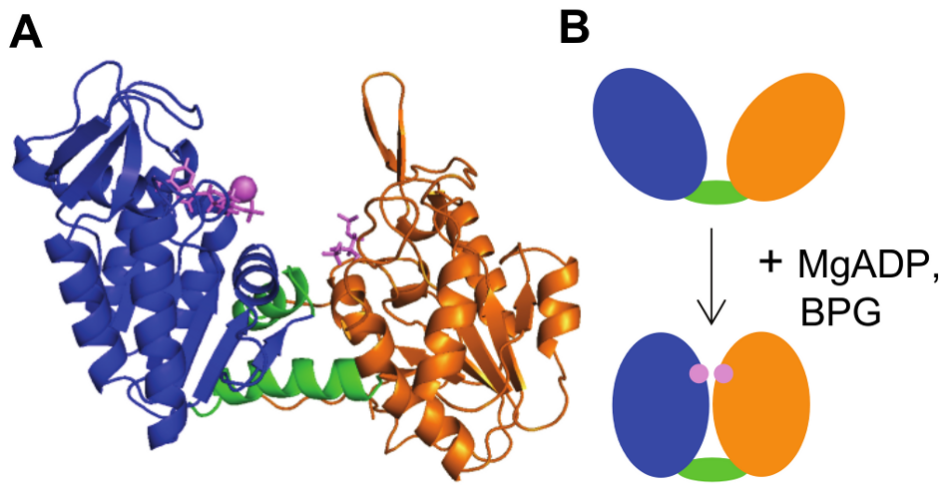
Structure of hPGK. (A) Cartoon representation of X-ray structure of hPGK. N-domain, orange; C-domain, blue; interdomain region, green; substrates (BPG and ADP), purple sticks; Mg ion, purple sphere. (B) The hinge bending mechanism of hPGK. Coloring as in (A). Substrate binding triggers a closure of the two domains, thereby bringing the two reaction partners into close proximity.

Crystal structures of PGK from numerous species have shown the enzyme in two distinct conformations: the open conformation 7–12, where the substrate binding sites are too far from each other (12–15 Å) for the phosphoryl transfer reaction to occur (Figure 1), and the closed conformation [13]–[15], where the substrates are proximal enough to allow nucleophilic attack. Thus, these end states are experimental evidence for a hinge bending motion of the enzyme that brings the substrates of this bimolecular reaction together. From combined crystallographic data and small angle X-ray scattering, it has been hypothesized that a spring loaded trap and release mechanism regulates the opening and closing of the domains [12]. By normal mode analysis of the open structure of PGK, Guilbert et al. [16] described three types of interdomain motions: hinge bending, twisting and a shear motion. Our previous Molecular Dynamics (MD) simulations showed that both the apo and the complex enzyme exhibit a small amplitude hinge bending type of motion on nanosecond scale, with the substrate binding changing the character of the motion and restraining the hinge bending [17]. These data put forward the notion of PGK being able to exhibit fast small amplitude hinge bending motions even in the apo state. However, full closure of the enzyme upon substrate binding to adopt the active conformation requires a large and directional hinge bending motion. This raises the question of how the signal of substrate binding penetrates to the interdomain region, where the conformational change happens, leading to closing/opening of the enzyme. Szabo et al. [18] in their combined mutational and kinetic experimental work identified amino acids that play a role in the communication between the substrate binding sites and the hinge region of the enzyme, in particular reported Arg38, Lys219, Asn336 and Glu343 as residues essential in domain closure.

A coherent signaling pathway from the binding sites to the hinge region of hPGK has, however, remained elusive. Computational methods that have been developed to describe allosteric communication are mostly based on coordinates, among others by following the correlated motions as observed in Molecular Dynamics (MD) trajectories [19]–[22] or Elastic Network models [23], [24]. However, typical timescales of allosteric transitions are in order of millisecond to seconds, while atomic simulations cover the femtosecond to microsecond range. Also, these methods focus on large-amplitude motions, while allosteric signal propagation is likely to involve pathways through the rather stiff protein core. Force Distribution Analysis (FDA) recently developed in our group [25], [26] is based on inter-atomic forces instead of coordinate changes and has proven useful in revealing the intramolecular communication pathways of the allosteric proteins MetJ [26] and Hsp90 [27]. FDA monitors the change in pairwise atomic forces within the protein structure upon ligand binding or other perturbations. The advantage of this force-based method over coordinate-based approaches such as Elastic Network Models [28]–[30] or Principle Component Analysis of MD trajectories is three-fold. First, forces within a protein upon perturbation equilibrate relatively fast as compared to the conformational change they trigger [31]. Secondly, pairwise forces are based on internal coordinates and thus do not require any fitting as opposed to coordinate-based observables such as atomic fluctuations. Thirdly, secondary structure elements such as helices or beta-sheets in the protein core are relatively rigid as compared to outer loops of a domain. Consequently, they typically feature small fluctuations hidden in an analysis of (correlated) motions or normal modes, and yet can propagate large forces upon slight hinging or twisting [26], [27]. We here show a connected allosteric pathway of hPGK originating from the two substrate binding sites and extending into the interdomain region involved in hinge bending. Our force distribution analysis identified two primary hinge points in a helix and a loop of the interdomain, the straightening of which causes closing and activation of hPGK. Residues previously identified to be crucial for the regulation of hPGK [18] are part of our computed force network, which also comprises other interesting candidates for point mutations to test our allosteric mechanism.

## Results/Discussion

### Characterization of the binding pockets

**Figure 2:**
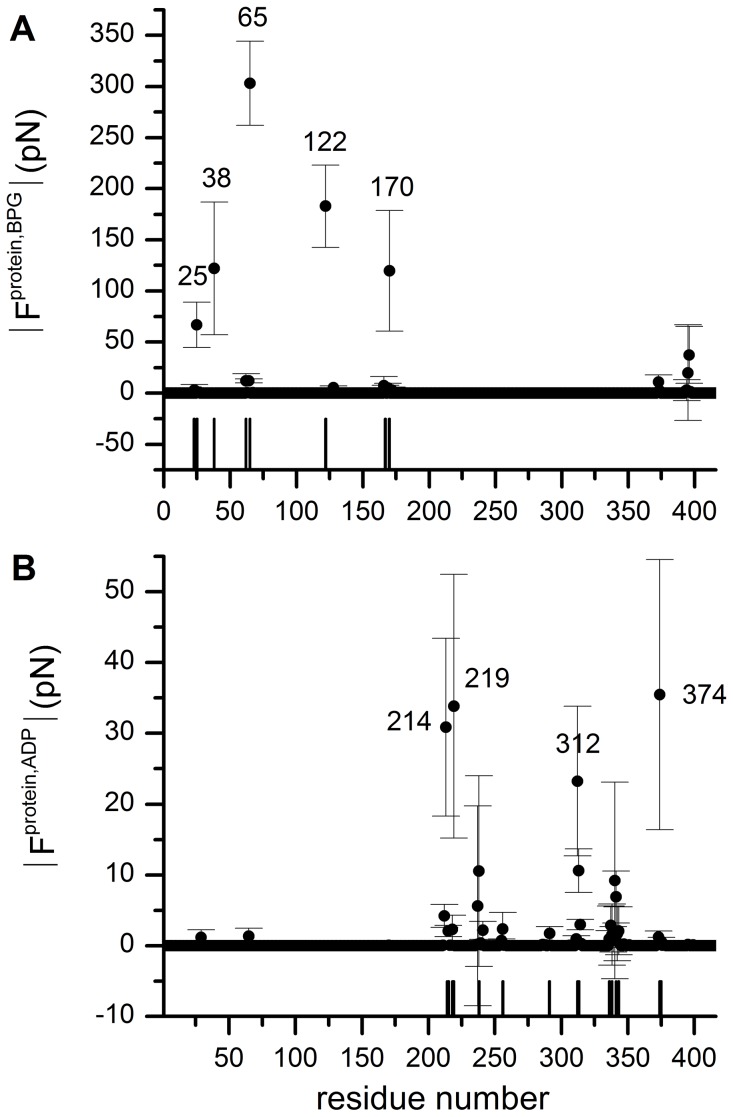
Forces exerted by the ligands on the protein. Absolute value of the averaged residue-wise forces between (A) BPG and hPGK residues, (B) ADP and hPGK residues. Experimentally identified binding residues are marked with vertical lines and labels for BPG (A) and ADP (B), respectively.

To characterize the strength of substrate binding to the hPGK complex, residue-wise forces, 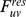, between the substrates and all protein amino acids were calculated by summing up forces *F_ij_* for all pairs of atoms *i* and *j* in residues *u* and *v*, where atoms *i*∈*u*, *j*∈*v*. The results presented in Figure 2 identify amino acids subjected to high forces upon ADP or BPG binding, which are in a very good agreement with the experimentally found binding residues [9], [12], [32], [33]. By comparing the absolute force magnitudes for the substrates, we observe that BPG exerts stronger forces on its binding residues. According to experimental binding measurements, BPG also shows a higher binding affinity for hPGK as compared to ADP [34]. Thus, here, the higher BPG-hPGK interaction forces, which are mostly attractive and of electrostatic nature, reflect steeper binding potentials, i.e. stronger binding. Indeed, we obtained a potential energy between BPG and hPGK of −835±3 kJ/mol, as opposed to −306±50 kJ/mol between MgADP and the protein from our MD simulations (Note that this is an only qualitative comparison as solvent and entropic contributions are missing in this rough estimation for a direct comparison to experiment).

**Figure 3:**
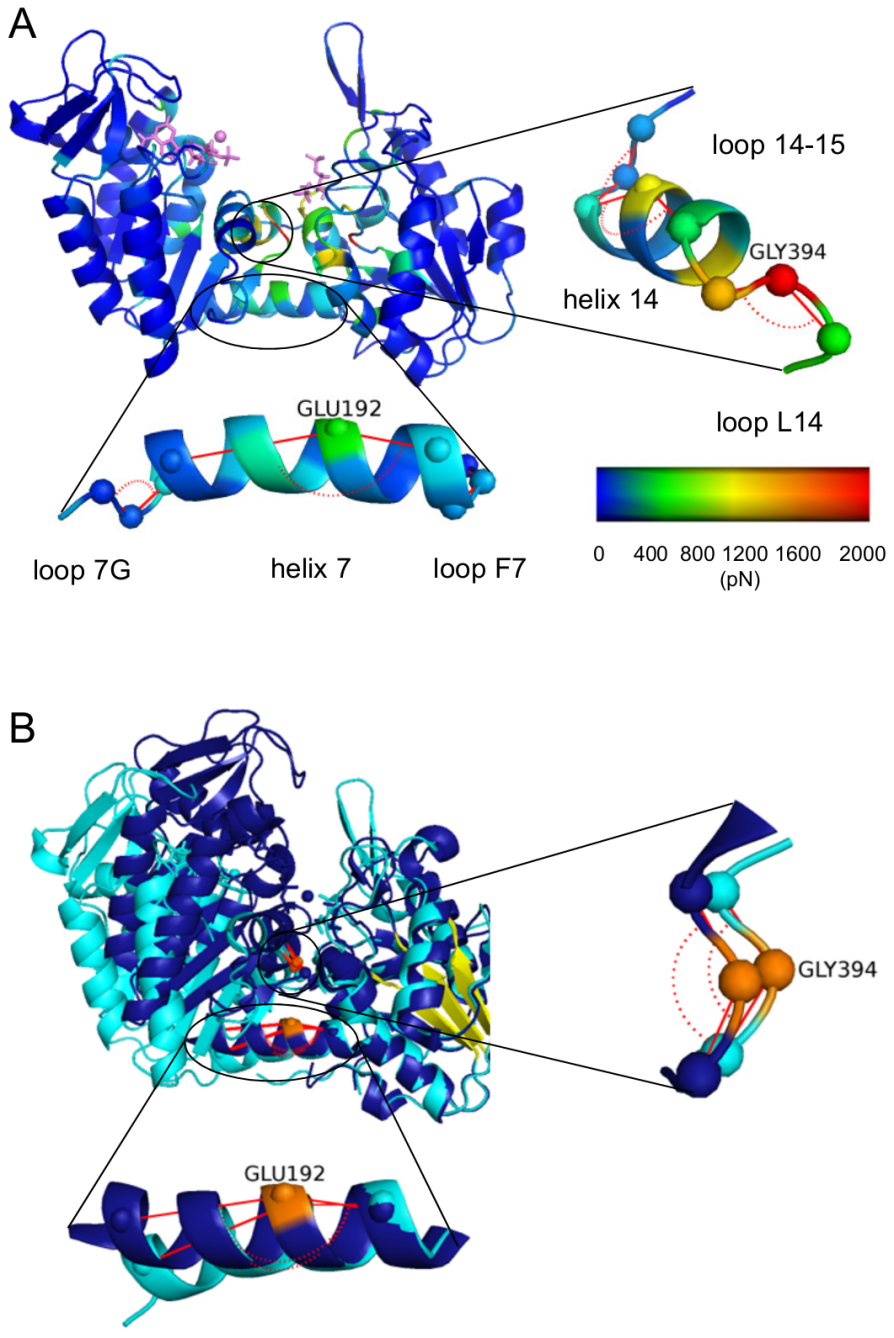
Bending residues of hPGK. (A) Color coded representation of atomic punctual stresses between apo and complex hPGK. Colors range from blue (minimum value) to red (maximum value). BPG and ADP are denoted by purple sticks, the Mg-ion by a purple sphere. Right: enlarged view of the interdomain region formed by helix 14 and loops L14, 14–15. Spheres show Cα atoms between which the bending angles (denoted by red lines) were calculated (Table 1). Bottom: enlarged view of interdomain region formed by helix 7 and loops F7, 7G. Spheres show Cα atoms between which the bending angles (denoted by red lines) were calculated. (B) Open (cyan) and closed (blue) X-ray structure of hPGK superimposed to the N-domain β-core (yellow). ADP and BPG are marked by sticks, the Mg-ion by a sphere. Enlarged views of the interdomain regions of loop L14 and helix 7. Orange spheres indicate the hinge points.

The difference in atomic pairwise forces, Δ*F_ij_* between the apo hPGK and the complex was calculated to analyze for the effect of perturbation caused by substrate binding. Summing up pairwise force changes sensed by an atom (
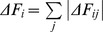
), the obtained atomic punctual stresses, Δ*F_i_*, were mapped on the three dimensional structure of hPGK (Figure 3A), with blue showing minimal and red maximal punctual stresses. By examining the nature of these forces we found that electrostatic interactions are playing the dominant role (see Suppl. Figure S1).

We observed an overall asymmetric pattern of stress in the two domains of hPGK. The BPG binding site shows greater punctual stresses than the ADP binding site, which can be interpreted as a consequence of the tighter binding of BPG compared to that of ADP (see above). Regions distant from the binding sites remain unperturbated by substrate binding. An exception is the interdomain region – in particular Glu192 in helix 7, Ser398/Glu400 in helix 14, and Gly394 in loop L14 – which exhibits a high degree of stress.

### Identification of hinge bending residues

We initiated the simulations of both the apo and ligand-bound state from the same experimental structure (complex, 2XE7), and observed the spontaneous opening of the two domains in the apo state, as expected, validating our MD setup. To study whether the environment of residues with high stress feature significant conformational changes by acting as a hinge point, angles formed by Cα atoms were calculated in the two interdomain regions (helix 7 and its adjacent loops F7 and 7G; helix 14 and its adjacent loops L14 and 14–15) for the apo and the complex systems (see Figure 3A right and bottom inserts). Table 1 shows the angle values averaged for the 9 MD trajectories and their differences between apo hPGK and complex. Upon substrate binding, loop L14 and helix 7 exhibit significant changes in their bending angles, namely −23 and −7.2 degrees, correspondingly, which indicates a flattening of these secondary structural elements. The other neighboring structural elements show only minor changes.

**Table 1:** Angle values in interdomain region.

	helix 7 (°) ∢188–192–199	loop F7 (°) ∢185–186–187	loop 7G (°) ∢201–202–203	helix 14 (°) ∢395–400–403	loop L14 (°) ∢393–394–395	loop 14–15 (°) ∢403–404–405
**apo**	153.6±1.9	127.6±0.2	113.4±0.9	140.0±0.6	97.4±2.8	101.7±0.6
**complex**	160.8±0.6	130.0±0.3	114.1±0.5	138.0±0.5	120.4±4.6	103.1±1.2
**apo -complex**	***−7.2±2.0***	−2.3±0.3	−0.7±1.00	2.0±0.8	***−23.0±5.4***	−1.4±1.3

Values of the angles defined between Cα atoms of given residues in interdomain region for apo and complex hPGK (Figure 3A). The angles are averages over 9 MD trajectories of each system.

The superposition of the open structure to the known catalytically active closed hPGK X-ray structures (PDB entry 2WZB [15] or 2WZC [15] or 2X13) corroborates our results: in all cases, the experimental structures feature a similar flattening of these secondary structural elements in the closed form with respect to the open form. As an example, Figure 3B shows the superposition of the open structure and 2WZB. This similarity also verifies the capacity of our simulations to capture those conformational rearrangements of the interdomain region, which facilitate the domain closure.

It can be seen in Figure 3B that by the “inverse bending” of helix 7, the C-domain moves towards the N-domain. Similarly, flattening of loop L14 provides space for the C-domain to get closer to the N-domain. According to Figure 3B, hinges of these movements are located at about residues Glu192 and Gly394, which are identical with the high-stress “hot spots” determined by FDA. The results of our angle analysis point out that these high stresses are accompanied by extensive conformational changes even in rigid secondary elements such as helix 7. Based on the available PGK sequences found in the ExPASy Molecular Biology Server [35], both residues are highly conserved, which underlines their likely role in domain allostery. In previous studies, Szabo et al. [36] showed that the side-chain of Glu192 is involved in the hinge motion, since its mutation led to substantial decrease of the catalytic efficiency. Furthermore, their calorimetric and thiol-reactivity studies showed that Glu192 is a key residue in maintaining the structural stability of the whole PGK molecule.

Instead of Glu192, previous studies – based on visual comparison of open and closed structures from different species [13], [37] or on MD simulations of these systems [17] – identified the N- and C-terminal loop of helix 7 as hinge points. The other hinge point, Gly394 loop L14, however, has been successfully identified [37], by visual inspection of the two X-ray structures of different species. Here FDA allowed to reveal the allosteric hot spot in the middle of helix 7, which was overlooked in previous studies based on conformational analyses of high amplitude motions.

The FDA results, however are coherent with the DynDom comparisons [39] of hPGK crystallized in open and closed conformations where the following hinge regions were suggested: Tyr195-Leu200 (helix 7), Leu211-Asp228 (helix 8) and Ser392-Gly394 (sheet βL-loopL14).

**Figure 4:**
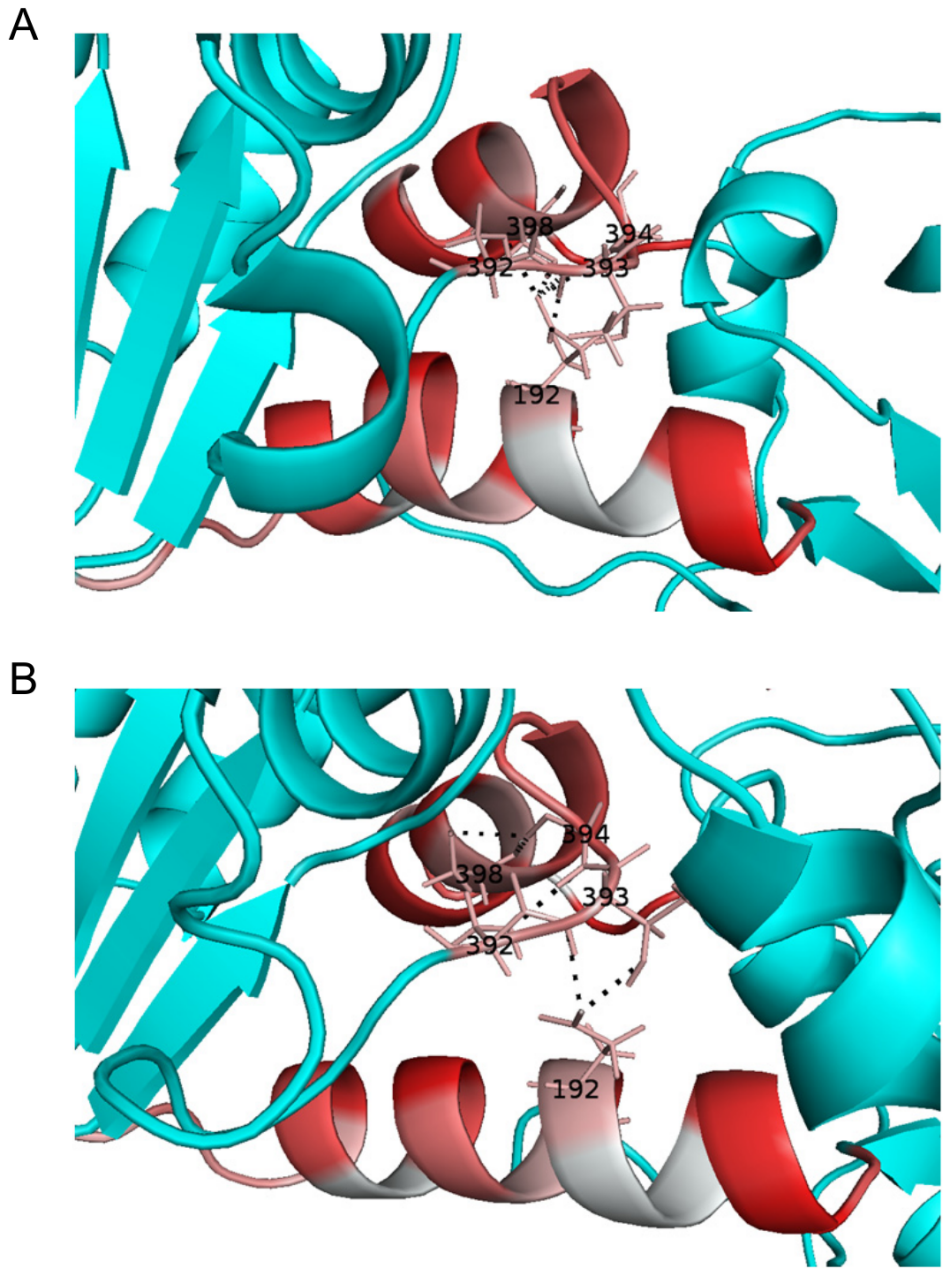
Hydrogen-bond network in hinge bending region of apo (A) and complexed (B) hPGK. Hydrophobic amino acids are shown in red, H-bonds are depicted with dashed lines.

Based on crystal structures of the complex and solution small angle x-ray scattering data Zerrad et al. [12] suggested a “spring loaded mechanism” for the domain movement of hPGK that is driven by the hydrophobic residues of the interdomain region. The open conformation is stabilized and thus favored by the burial of these hydrophobic residues, while a closing of the domains entails their exposure, counteracted by a stabilization through ionic interactions to allow catalysis. We can extend this scenario by the additional role of the hydrogen bond network in the hinge region. Figure 4 shows both the hydrophobic amino acids (in red) of the interdomain region and the hydrogen bonds (enlisted also in Suppl. Table S2) formed by the hinge points for apo and complex forms of the enzyme. Upon complexation, the hydrogen bond network weakens between Glu192 and adjacent residues (Ser392, Thr393, Gly394 and Ser398) and is only partially substituted (Ser392-Gly394 and Gly 394-Ser398), which we hypothesize to cause the subsequent extensive flattening of helix 7. This suggests the hydrophobic area exposure and the weakened H-bond network of the interdomain residues to work in concert, i.e. the burial of hydrophobic residues in the apo form and the stronger hydrogen bonds jointly “pull” the hinge of helix 7 towards the interdomain region and curve the helix. As our analysis cannot identify causalities of the observed effects, similarly, the helix hinging could reversely effect substrate binding sites and substrate or product release.

### Identification of the signaling pathway

The distance between the substrates and the identified hinges poses the question of the pathway of the long-range communication between these two regions, i.e. the signaling pathways through which stress propagates from binding residues towards the functionally important interdomain region.

**Figure 5:**
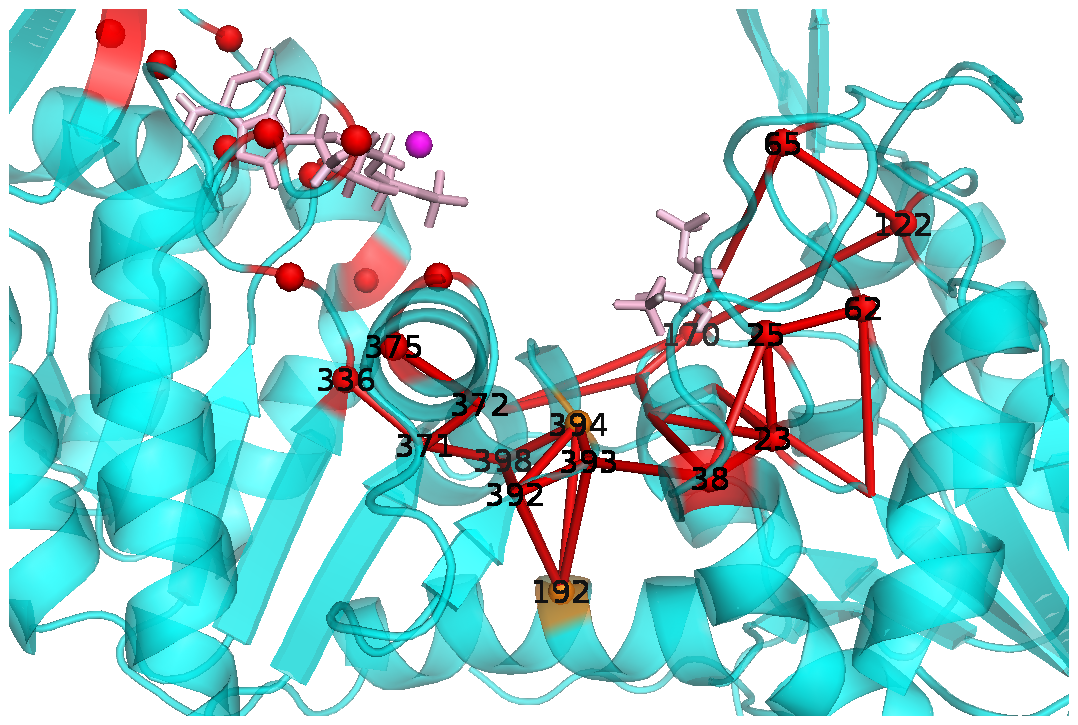
Force distribution in hPGK. Network-like representation of significant changes in inter-atomic forces. Red edges connect residue pairs having inter-atomic forces with |ΔF*_ij_*| >90 pN. Red spheres indicate the substrate binding residues. BPG and ADP are denoted with purple sticks while Mg-ion is marked as a purple sphere. The orange spheres mark the two hinge points Glu192 and Gly394.

Figure 5 displays the largest connected network of pronounced atomic pairwise force changes. Edges connect C_α_ atoms of two residues with an inter-atomic force difference |ΔF*_ij_*|>90 pN. While all of the BPG binding residues are involved in the signal transmission, only two of the ADP binding residues transmit the perturbation. Since BPG exerts higher forces on its binding residues, these residues are capable of propagating the perturbation in different directions throughout the structure of the protein. While the weaker binding of ADP to the protein results in a low number of binding residues being involved in signal transmission. Figure 5 indicates that the two hinge points, Glu192 and Gly394, are included in the network, suggesting the remote binding of the substrates to perturb the force distribution at the hinges, a clear evidence of allostery. The hinges are perturbed via several force distribution pathways stemming from binding residues of both substrates, indicating that hinges detect the effects of both substrates. Furthermore, the hinges act in concert, since they are directly connected in the path. The indirect communication between the two substrates of hPGK through this stress transmission path, jointly with a direct electrostatic repulsion between the substrates of 92±38 pN (between MgADP and BPG, obtained from FDA), can explain the cross-talk of the two molecules in terms of their affinity for hPGK [6] and in terms of their joint action to trigger the hPGK conformational change [38], [40].

Tracking down the transmission path of BPG, we can note that the BPG binding residues form a tight interaction network around the substrate and are further passing down the force signal to Arg38 and Arg170. From Arg38, stress propagates through Thr393 to both hinges, Glu192 and Gly394. Thus, the signaling pathway of the BPG effect towards the hinges is: *BPG→BPG binding residues* (Arg122, Arg65, Arg170, His62, Asp23, Asn25, Arg38)*→Thr393→hinge residues* (Glu192, Gly394). Similarly, tracking down the stress propagation path due to ADP binding, we can note that only two binding residues, Asn336 and Thr375 transmit the effect of ADP. From Asn336 the stress propagates through Gly371, Ser398 and Ser392 to reach the two hinges, Glu192 and Gly394. The stress originating in Thr375 is transmitted by Gly372 (which in our previous study, based on conformational analysis, was identified as a hinge point) and then follows the same path. Thus, the signaling pathway of the ADP effect towards the hinges is: *ADP→ADP binding residues* (Asn336, Thr375)*→Gly371, Gly372→Ser398, Ser392→hinge residues* (Glu192, Gly394).

### Conclusion

Previous structural and dynamical analyses of PGK allostery have allowed unprecedented insight into the large conformational changes required for phosphoryl transfer catalysis. We here could reconcile and complete the picture of a signal transduction pathway from the ADP and BPG binding sites to the interdomain hinges Glu192 and Gly394, using our novel force distribution analysis. Our allosteric pathway overlaps with a previously identified transmission path, which however was restricted to a single hinge in sheet L [18]. While previously identified key residues involved in PGK domain closure [1], [18], [34], [36] are part of this pathway, underlining their functional relevance, residues Gly371, Gly372 and Gly394 are residues newly uncovered as critical allosteric spots. We hypothesize that an addition of a sidechain to these residues would abolish or measurably alter hPGK allostery, and thus suggest them as interesting candidates for future mutational studies.

## Methods

### Molecular Dynamics (MD) simulations

MD simulations were carried out on apo hPGK and on its natural ternary complex: ADP*BPG*Mg*hPGK (complex). The starting structures were derived from the crystal structure of hPGK (PDB entry 2XE7) complexed with ADP and 3-phospho-glycerate (PG). The apo structure was built by removing ADP and PG from the 2XE7 structure. Starting all simulations from the 2XE7 structure instead of two different experimental structures for complex and apo states allowed to validate our MD setup by tracking the unbiased relaxation from the complex to the more open apo state and to measure force differences (see below) between the two states only due to this allosteric conformational change and not due to any potential additional differences in starting structures because of crystallization conditions or crystal packing. For the ternary complex, an extra phosphate group and a Mg-ion were placed into the 2XE7 structure based on the MgADP bound structure (PDB entry 1PHP) [32] of *B. Stearothermophilus* PGK using the Schrödinger-Maestro program [41]. The extra phosphate group was covalently attached to the 1-carboxyl group of PG and coordinated by Arg38 and a water molecule via non-bonded interactions.

The initial three N-terminal residues missing in the crystal structure were modeled using the Schrödinger-Maestro program. Coordinates for the N-terminal loop and loops showing extraordinarily high B-factors were optimized by the Modloop web server, version r181M [42], [43]. The water molecules present in the crystal structure were retained around the binding sites.

MD simulations were performed with GROMACS 4.5.1 [44] using the CHARMM all-atom parameter set 27 [45]. Both systems, apo hPGK and the ternary complex, were immersed in a rhombic dodecahedron box of TIP3P water [46] with vector length 100 Å with a distance of 12 Å between the protein surface and the box face. The boxes were replicated by periodic boundary conditions. Sodium and chloride ions corresponding to a physiological ion strength of 120 mM were added. Additional chloride or sodium counterions were added to achieve a neutral net charge of the apo and complex systems, respectively.

The real space summation of electrostatic interactions was truncated at 12 Å, and the Particle Mesh Ewald (PME) [47] method was used to calculate the electrostatic interactions beyond 12 Å with a maximum grid spacing of 1 Å and an order of 6. The width of Gaussian distribution was set to 0.34 Å^−1^. Van der Waals interactions were calculated using a cut-off of 12 Å.

The solvated systems were energy minimized by the following procedure: the steepest descent algorithm was used first with harmonic constraints applied to heavy protein atoms to achieve smooth minimization. The harmonic force constant was decreased every 100 steps, adopting the values 40 000, 10 000, 1 000 and 100 kJmol^−1^ nm^−2^. Then unconstrained minimization was applied for 100 steps with steepest descent, followed by 1000 steps of conjugate gradient algorithm.

The energy minimization was followed by a 5 ns MD simulation with harmonic constraints on protein heavy atoms with a force constant of 1000 kJmol^−1^ nm^−2^ to equilibrate water and ions around the protein. An unconstrained MD simulation of 3 ns length was performed to equilibrate the whole system.

For each system, 9 independent 50 ns production MD simulations were performed totaling 900 ns of simulation time. An integration time step of 2 fs was used. The coordinates of the trajectories were saved every 50 ps. For each simulation new random velocities were generated and different initial frames were used to enhance conformational sampling. Starting frames were chosen from the second half of the first MD simulation for subsequent simulations. Simulations were carried out in the NPT ensemble. The temperature was kept constant at 300 K using the Nosé-Hoover thermostat [48] with a time constant of 0.4 ps. The pressure was set to 1 bar using isotropic coupling to the Parrinello-Rahman barostat [49] with a time constant of 1 ps and a compressibility of 4.5*10^−5^ bar^−1^. All bonds were constrained using the LINCS [50] algorithm. The stability of the simulation is shown by Suppl. Figure S2.

### Force distribution analysis

We used the FDA extension [51] for GROMACS 4.5.3 to write out forces, *F_ij_*, between each atom pair *i* and *j* as calculated during our MD simulations. Forces were computed between all atom pairs within the cut off range for each frame of the MD simulation, and included all interaction types (bonds, angles, dihedrals, electrostatic and Lennard Jones interactions). Residue-wise forces, 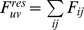, were introduced by summing up forces *F_ij_* for all pairs of residues *u* and *v*, where atoms *i*∈*u*, *j*∈*v*, which in our case were used only for the characterization of the binding pocket.

Since pair-wise force vectors are affected by rotation and translation of the system, for subsequent analysis, the norm of the force was used, with opposite signs assigned to attractive and repulsive forces [26]. Forces for each system were averaged over all nine equilibrium trajectories, each 50 ns in length, to achieve converged averages. Differences between averaged pairwise forces (Δ*F_ij_*) of the apo and complex form of hPGK were then calculated to describe the perturbation of substrate binding. While we observed positive and negative pair-wise forces to similar extent, for both, *F_ij_* and Δ*F_ij_*, implying a balance of repulsion and attraction within the protein at equilibrium, we could not infer any relevance of the sign of the forces for the allosteric mechanism, so that we restricted the analysis presented in the Results section to the absolute values only for the sake of clarity. Atomic punctual stress was defined as the absolute sum of force differences sensed by a single atom: 
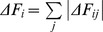
. Here, absolute forces are summed up, because the sum of positive and negative Δ*F_ij_* values is by definition zero over time, i.e. are balanced, resulting in the absence of any net translation of the system during the MD simulation time. The atomic punctual stress has been defined previously [51] and here serves as an easily accessible measure of the perturbation upon ligand binding. It is similar but not equal to the virial stress and other more complex definitions of local stresses. [52], [53]. The convergence of force are shown on Suppl. Figure S3. For the identification of pathways of stress propagation, the largest network of connected atomic pairwise force differences between apo and complex hPGK beyond a cut off value was determined using a vertex count algorithm [26]. The cut off for force differences was chosen such that a consecutive path originating from the substrate binding sites was obtained [27]. A larger cut off than 90 pN resulted in only small vertex counts, i.e. a broken path, while a smaller cutoff resulted in additional networks at distant unconnected sites, indicative of noise in the forces.

## Supporting Information

Figure S1 **Nature of the long-range forces.** Absolute value of atom-wise forces (A) Apo- (B) complexed form of hPGK. (C) Difference of the forces between apo and complexed form of hPGK. Black represents Coulomb, green van der Waals forces.
(TIF)

Figure S2 **Overall behavior of hPGK under the simulation.** (A) Distance between the center of masses of the N- and C-domains during the simulation for the apo (cyan) and complexed (blue) form of hPGK. (B) Snapshot of one of the most open apo structure (cyan) and the most closed complexed structure (blue) observed in the simulations. The structures are superimposed to the N-domain β-core (yellow). ADP and BPG are marked by sticks, the Mg-ion by a sphere. Orange spheres indicate the hinge points.
(TIF)

Figure S3 **Convergence plot of forces.** An example of the convergence of pairwise forces between Met355:O and Val359:N of the apo (solid line) and complex (dotted line) form of hPGK.
(TIF)

Table S1 **Secondary structural elements of hPGK.**
(DOCX)

Table S2 **H-bonds and their distances in Å in the hinge region of hPGK.**
(DOCX)
