# Supplementary figures and images for: An Allosteric Signaling Pathway of Human 3-Phosphoglycerate Kinase from Force Distribution Analysis
Source: PLoS Comput Biol. 2014 Jan 23;10(1):e1003444. doi: 10.1371/journal.pcbi.1003444 (PMC3900376; doi:10.1371/journal.pcbi.1003444)

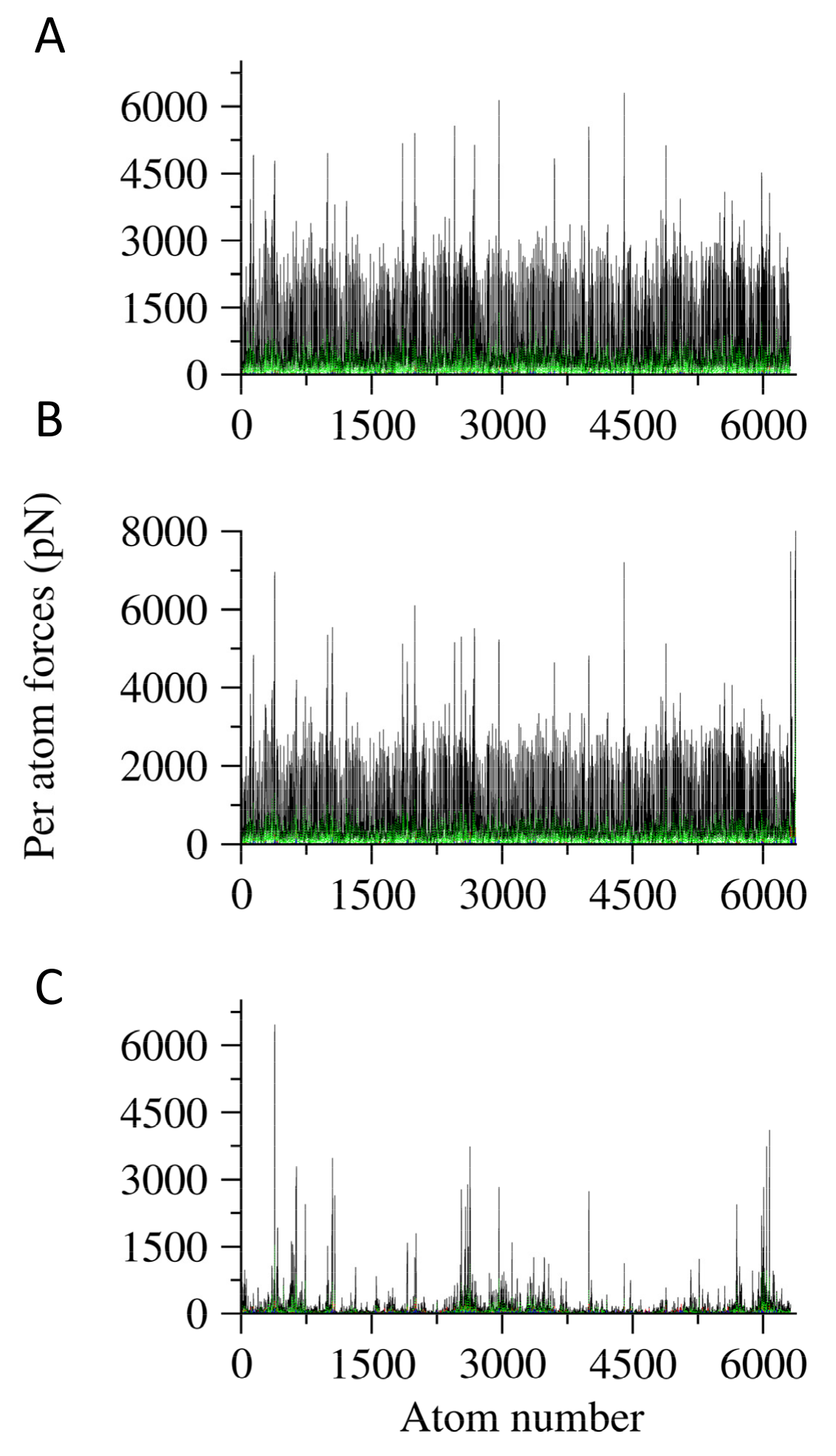

Supplement: Figure S1 — Nature of the long-range forces. Absolute value of atom-wise forces (A) Apo- (B) complexed form of hPGK. (C) Difference of the forces between apo and complexed form of hPGK. Black represents Coulomb, green van der Waals forces. (TIF) [file pcbi.1003444.s001.tif]

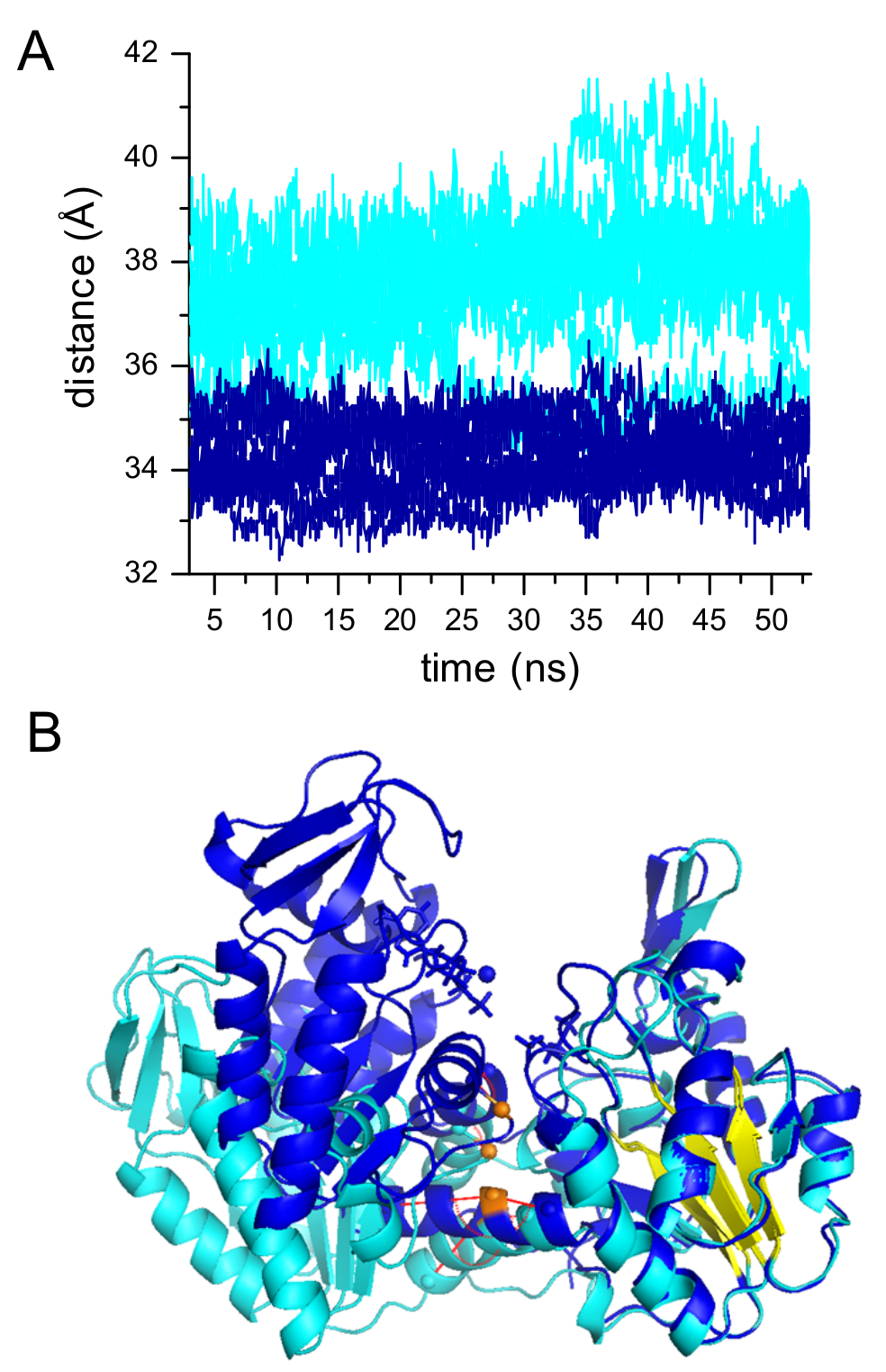

Supplement: Figure S2 — Overall behavior of hPGK under the simulation. (A) Distance between the center of masses of the N- and C-domains during the simulation for the apo (cyan) and complexed (blue) form of hPGK. (B) Snapshot of one of the most open apo structure (cyan) and the most closed complexed structure (blue) observed in the simulations. The structures are superimposed to the N-domain β-core (yellow). ADP and BPG are marked by sticks, the Mg-ion by a sphere. Orange spheres indicate the hinge points. (TIF) [file pcbi.1003444.s002.tif]
