# Supplementary material for: An Allosteric Signaling Pathway of Human 3-Phosphoglycerate Kinase from Force Distribution Analysis
Source: PLoS Comput Biol. 2014 Jan 23;10(1):e1003444. doi: 10.1371/journal.pcbi.1003444 (PMC3900376; doi:10.1371/journal.pcbi.1003444)
Supplement: Table S1 — Secondary structural elements of hPGK. (DOCX) [file pcbi.1003444.s004.docx]

| Residue number | Name |  |
| --- | --- | --- |
| 17-22 | A |  |
| 36-40 | 1a |  |
| 41-52 | 1b |  |
| 56-61 | B |  |
| 77-89 | 2 |  |
| 91-96 | C |  |
| 101-109 | 3 | N-domain |
| 114-119 | D |  |
| 124-128 | α extra |  |
| 129-134 | m |  |
| 136-141 | n |  |
| 144-155 | 4 |  |
| 158-163 | E |  |
| 165-169 | 5 |  |
| 173-178 | 6 |  |
| 182-187 | F |  |
| 189-202 | 7 |  |
| 207-212 | G |  |
| 218-228 | 8 |  |
| 231-236 | H |  |
| 239-249 | 9 |  |
| 261-264 | 10a |  |
| 266-275 | 10b |  |
| 277-282 | l |  |
| 283-289 | o | C-domain |
| 296-301 | p |  |
| 310-315 | q |  |
| 317-330 | 11 |  |
| 332-336 | J |  |
| 348-365 | 12 |  |
| 367-371 | K |  |
| 373-380 | 13 |  |
| 388-392 | L |  |
| 396-404 | 14 | N-domain |
| 408-414 | 15 |  |
