# Supplementary material for: An Allosteric Signaling Pathway of Human 3-Phosphoglycerate Kinase from Force Distribution Analysis
Source: PLoS Comput Biol. 2014 Jan 23;10(1):e1003444. doi: 10.1371/journal.pcbi.1003444 (PMC3900376; doi:10.1371/journal.pcbi.1003444)
Supplement: Table S2 — H-bonds and their distances in Å in the hinge region of hPGK. (DOCX) [file pcbi.1003444.s005.docx]

| apo | GLU192/OE1 | SER392/OG | 2.59 |
| --- | --- | --- | --- |
|  | GLU192/OE1 | THR393/N | 3.48 |
|  | GLU192/OE2 | THR393/N | 2.76 |
|  | GLU192/OE1 | GLY394/N | 3.23 |
|  | GLU192/OE1 | SER398/OG | 2.61 |
| holo | GLU192/OE1 | THR393/N | 2.76 |
|  | GLU192/OE1 | THR393/OG1 | 2.84 |
|  | SER392/OG | GLY394/N | 3.09 |
|  | GLY394/O | SER398/N | 2.97 |
|  | GLY394/O | SER398/OG | 2.88 |
